# Facilitating engagement of universal school-based digital mental health solutions through user experience: A qualitative exploration

**DOI:** 10.3389/fdgth.2023.1040739

**Published:** 2023-03-22

**Authors:** Erfan Badawi, Constantinos K. Coursaris, Sylvain Sénécal, Pierre-Majorique Léger

**Affiliations:** ^1^Department of Information Technologies, HEC Montréal, Montréal, QC, Canada; ^2^Department of Marketing, HEC Montréal, Montréal, QC, Canada

**Keywords:** mental health, digital mental health interventions, adolescents, high school, qualitative, exploration, user experience, engagement

## Abstract

Digital mental health intervention (DMHI) programs offered in schools present a readily-accessible and flexible means for educating, empowering, and supporting adolescents in maintaining a balanced mental health, especially during uncertain and stressful times such as the COVID-19 pandemic. Recent studies indicate that the effectiveness of DMHI programs in improving students’ mental well-being and in preventing from their mental health complications depends on the users’ engagement. This study focuses on identifying the user experience factors that can facilitate user engagement with universal school-based DMHI programs (i.e., the DMHI programs delivered to the students regardless of their mental health risks or conditions). To identify said factors, we sought to gain a deeper understanding of perceptions, opinions, and preferences of actual end-users (i.e., the adolescents) regarding their experiences with both digital and non-digital mental health resources. Specifically, interviews were conducted with two participant groups to uncover the reasons that could lead the adolescents to better engage with school-based DMHI programs, as well as the shortcomings that could prevent that from happening: (a) adolescent users who had either a high or a low level of engagement with universal DMHI programs of a specific school-based digital mental health solution; and (b) adolescents who had voluntarily used non-digital or non-school-based digital mental health resources for purposes other than treatment. Through a thematic analysis of interview data, the most important (or primary) and the additionally desirable (or secondary) factors that could lead to a higher engagement level for school-based DMHI programs were identified. Lastly, using the evidence gathered from our interviews, specific recommendations are proposed that could help in targeting each identified engagement factor and in increasing the likelihood that school-based DMHI programs achieve their desired outcome for high school students.

## 1. Introduction

The adverse effects of the coronavirus disease (COVID-19) pandemic on public health have invited renewed attention toward adolescents’ access to mental health resources, support, and education. As recent public health studies and surveys on mental health in North America indicate, adolescent students are in an alarming mental health condition, especially due to lack of in-person interaction and the uncertainties resulting from measures such as lockdowns and closures of schools that were intended for preventing the spread of COVID-19 (1, 2). The decline in mental health conditions has not only affected the overall health of adolescents, but also has caused adverse social outcomes, such as lower school grades and difficulty in making friends (3). Even though evidence strongly suggests that adolescents’ mental health requires immediate attention, not only do the younger generations lack access to both mental health resources and education (4), but “mental health” is still a topic that cannot be openly discussed. Research shows that there are still generally negative perceptions and stigma associated with adolescents seeking professional help for mental health concerns and problems (5). Therefore, schools can play a key role in addressing these problems by acting as the most accessible (due to costs, transportation and other factors involved) and the most acceptable (due to the stigma associated with traditional mental health treatment) mental health resource centers and educators (6, 7).

Evidence has shown that schools are environments where universally delivered mental health intervention programs can be effective in achieving their goals (e.g., anxiety and depression prevention) and in improving adolescents’ and children's overall mental well-being (8–10). In contrast to the “selective” and “indicated” types of mental health intervention programs—collectively referred to as “targeted” programs—which high schools design for more at-risk groups or individuals who show symptoms of a mental health disorder, “universal” mental health intervention programs are designed for and delivered to either all the students of specific grades or throughout the school (11). Therefore, compared to the targeted methods, universally delivered programs are not only more accessible, more cost-effective, and much easier to implement (12), but also are perceived to be much less stigmatizing since the individual students are neither singled-out nor forced to participate in a mental health program (11). Furthermore, by universally delivering a digital mental health intervention (DMHI), schools can allow their students to have a costless, flexible, and readily available access to a collection of mental health-related learning activities, practices, and other resources such as mental health support services (13).

Digital solutions (delivered *via* online platforms, mobile and wearable digital devices, and smartphone and computer applications), when employed as the primary or the supplementary method of delivering mental health intervention programs, provide crucial advantages over non-digital methods of intervention; these include reduced stigma associated with receiving or using a mental health support system, more privacy and anonymity for students, less training and preparation required for staff, and more flexibility and consistency in delivering mental health resources to students (14). The benefits resulting from universal school-based DMHI programs could potentially lead to more student engagement than the non-digital programs and could maintain the interest and involvement of these adolescent users throughout the intervention (14).

User engagement with digital technologies intended for interventions that promote self-management, self-monitoring, and behavior changes—similar to the features, qualities, and objectives of high schools with universal DMHI programs—has been conceptualized by Perski et al. (15), through an integrative definition. This conceptualization, which is based on a systematic review of 117 qualitative studies about engagement with digital intervention methods, is as follows: engagement is a dynamic process (i.e., it varies within individuals over time) underpinned not only by behaviors (such as the amount, frequency, and duration of use), but also by the subjective experiences of the users, within their contexts of use (15). Recent studies have all signified that it is vital to focus on improving the users’ engagement with DMHIs. Findings from a scoping review of 16 studies on user engagement with DMHIs in “nonclinical” settings (i.e., not designed for actual patients or persons undergoing a treatment) have indicated that for DMHIs to be effective for nonclinical users in improving their psychological well-being, optimal user engagement is critical (16). Also, results from a recent narrative review of 35 studies pertaining to the effects of engagement with DMHIs have revealed that, regardless of the types of intervention or the mental health condition of the target users, higher levels of user engagement with DMHIs were associated with moderate improvements in mental health outcomes for the users (17).

In our review of academic literature, we did not find recent research that explored the perspective of high school students to identify the factors that could lead to a more engaging user experience with school-based DMHI programs. The studies included in the reviews mentioned above were primarily concerning adults aged 18 and above, and outside the school environment; specifically, there were only five studies that included school-aged children and adolescents while none had particularly focused on adolescents or high school students. In a less recent systematic review of relevant literature concerning adolescents, researchers had also invited similar attention towards user engagement with DMHIs; in their review of school-based and college-based DMHI programs for young people aged 12–25, Clarke et al. (18) have concluded that DMHIs have positive impacts on adolescents’ mental health and well-being, but low levels of engagement and program dropouts have been significant issues affecting many past interventions.

To investigate what factors could facilitate adolescents’ engagement with school-based DMHIs, we sought to better understand adolescents’ subjective experiences, opinions and preferences surrounding digital mental health resources in general, and to uncover their needs, expectations and desires from universal school-based DMHI programs. To focus our efforts on this exploration, specific research questions were identified:

RQ1: What are the factors related to user experience of universal school-based digital mental health solutions that drive the engagement of frequent adolescent users with DMHI programs?

RQ2: The absence of which factors related to user experience of universal school-based digital mental health solutions leads to a lack of engagement with DMHI programs for their infrequent adolescent users?

RQ3: What factors related to user experience of universal school-based digital mental health solutions can facilitate high school students’ engagement with DMHI programs?

To address the first and second research questions (i.e., RQ1–2), we conducted in-depth investigations into the experiences, opinions, and preferences of actual users of universal school-based DMHI programs. We focused on these users, calling them the main participant groups of our study, in order to identify which addressed or unmet needs, expectations and desires had resulted in their increased engagement or disengagement with DMHI programs. Overall, the main participants interviewed comprised eight users, half of which were chosen because they had a considerably high engagement level with universal school-based DMHI programs, and the other half chosen due to their considerably low engagement level.

To answer the third research question (i.e., RQ3 which is the central research question of this study), data gathered from the main set and a supporting set of interviews was used to identify relevant user experience factors shared among all interview participants. A supporting set of interviews was conducted in order to account for relevant past experiences with other mental health resources that could influence user engagement with universal school-based DMHI programs. To this end, we recruited adolescents who were likely to become potential future users of DMHI programs—if their schools were to offer them universally to their students—so that their relevant experiences, opinions, and preferences pertaining to school-based DMHI programs could be investigated. Overall, and as part of the supporting set, eight adolescents were interviewed, all of whom had prior relevant experiences with either non-school based digital mental health resources (e.g., stress-relief or sleep aid applications) or non-digital mental health resources (e.g., in-person yoga, mindfulness or meditation classes), all in nonclinical settings and through their voluntary use or participation.

## 2. Methods

### 2.1. Context

To answer our research questions, we conducted exploratory interviews with adolescent users of universal school-based DMHI programs as well as users who had relevant experiences with other mental health resources (either non-digital or non-school-based digital resources) in nonclinical settings. Individual semi-structured interviews were conducted as their benefits heavily outweighed structured or non-structured interviews, as well as interviews with more than one participant; semi-structured interviews would provide us more flexibility in our exploration and more opportunities for probing, thus helping us uncover more insights into individual experiences and more evidence regarding the factors that contribute to user engagement than the other methods (19).

For determining a sampling method for our main participant groups (i.e., users with a considerably high or low level of engagement with universal school-based DMHI programs), Perski et al.'s (15) conceptual framework for engagement with digital interventions was used: “User Engagement” is conceptualized as a dynamic process and is defined as a bidimensional construct relying both on the usage behavior and user's subjective experience, within the context of use (15). This framework focused our exploratory efforts on the two extremes of the usage behavior spectrum: only users who had either high-frequency or low-frequency usage, and on one specific local digital mental health solution.

The participants were chosen from the users of a single specific local solution in order to limit major discrepancies in the settings pertaining to DMHI programs’ contexts of use that could have introduced contextual differences in the users’ perceived experiences and opinions; these settings, according to Perski et al.'s framework (15), would be underpinned by the users’ social environment (e.g., culture, social norms, and media) and physical environment (e.g., location, healthcare system and policy, access to high-speed internet and hardware). In addition, since according to the framework, the optimal level of engagement (or *optimal dose*) at which an intervention program would be effective is specific to and defined for a particular digital solution, a single specific digital mental health solution was chosen and focused on in order to investigate the factors that could have led to a user engagement level that is significantly lower or higher than this solution's optimal dose.

#### 2.1.1. Description of the chosen local digital mental health solution

The specific digital mental health solution that was selected for our study, which for the purpose of confidentiality will remain unnamed, was at the time of our study offering universal DMHI programs to two high schools (a private school and a public school) located in southwestern Quebec, Canada. This solution consisted of a web-based platform that provided weekly- and monthly-themed mental health intervention programs throughout the schoolyear for students who would voluntarily register for them. The solution's DMHI programs that were designed with and specifically for the two high schools were focused on demystifying “mental health” for students, reducing their psychological distress, and improving their overall well-being. Each of these DMHI programs consisted of a variety of activities presented within a gamified, step-by-step setting (i.e., completion of each activity awards points to the participating student); the platform also provided a dashboard for students so they could monitor their progress and performance, and leaderboards for comparing their performance statistics with those of other peers. Each program's activities included educational presentations (including readings and videos), recreational slides, quizzes, etc. that were all related to the specific theme of the weekly or monthly program, such as “self-acceptance”, “anxiety”, and “physical health”.

### 2.2. Recruitment and participants

For the main set of interviews, and through purposive sampling, we targeted adolescents who were studying at one of our two targeted high schools, and who had a noticeably high or low level of engagement with the chosen digital mental health solution. For the supporting set of interviews, we targeted adolescents residing in Canada who had voluntarily used or followed non-digital mental health resources (such as yoga, meditation, and mindfulness classes) or non-school-based digital mental health resources (such as digital tools and applications for anxiety- and stress-relief, sleep aid, and mental focus). The research protocol and interview guides for each participant group were submitted along with other necessary documents for the approval of our institution's Research Ethics Board (REB). In order to receive the informed consent for participation in our research directly from adolescents and interview them without an adult or guardian being present (as their presence alone could have influenced our discussions), our data collection was restricted^1^ to focus only on adolescents aged 14 and above (equivalent to high school students in grades 9–11 in Quebec, Canada).

After receiving the approval certificate from REB, the recruitment process commenced in late spring 2021, close to the local high schools’ final examination period. First, the directors of the selected digital mental health solution were contacted in order to find potential participants for the main set of interviews based on their solution's indicators for engagement. These indicators accounted for cumulative time and number of logins, number of completed activities and sessions, and the number of days passed since registration for each user; users fell into one of two groups: (a) the top 5% of students aged 14 and above with the best indicators for engagement were classified as “frequent users” of the solution's DMHI programs; and (b) the top 5% of students aged 14 and above with the worst indicators for engagement were classified as “infrequent users” of DMHI programs. For each of these two user groups, 33 potential participants met the inclusion criteria.

The search for potential participants that would meet the criteria set for the supporting groups (i.e., having past experiences with non-school based digital mental health resources or with non-digital mental health resources) was carried out in parallel with the search for main participants. The methods used for finding supporting participants consisted of contacting a network of local high schools to send our invitation letters to their students and posting “Call for participants” messages on social media platforms. All the potential participants were checked for good recollection of their experience with resources they had used; this was a necessary criterion for participating in our interviews, for which the selected participants would receive a fixed monetary compensation (CA$25).

Eight individuals were recruited for our main participant groups, who all self-identified as female comprising (a) four frequent users, two from the same private school and the other two from the same public school; in each of these high schools, one student was in Grade 9 and the other in Grade 10; and (b) four infrequent users all of whom were studying at the same private school; only one participant from this group was in Grade 10, while the other three were in Grade 9. Overall, out of the eight participants in the main set of interviews, five (62.5%) were studying in Grade 9 and others (37.5%) were in Grade 10; two students were studying at a public high school (25%) and others, at a private high school (75%).

In addition, eight adolescents were recruited for our supporting set of interviews; half were students at public and the other half at private high schools. Most participants identified themselves as female (75%), and only two as male (25%). Also, four adolescents (50%) recruited for these interviews met both criteria and responded to our questions about their experiences with both digital and non-digital mental health resources during the interviews of slightly extended duration. Overall, six adolescents (a) met the criteria for non-digital mental health resources: three of these adolescents were at public high schools where they had taken in-person, non-mandatory mental health courses; the other three were at private high schools, all of whom pursued mental health practices such as yoga and meditation outside the school environment and of their own accord; and (b) had voluntary experiences (without an intervention from a professional or organization) with non-school-based digital mental health resources for different reasons such as stress- or anxiety-relief, increasing their focus or sleep quality; half of these adolescents (3 out of 6) were students at private schools and the other half at public schools.

Combining the data from participants recruited for all groups resulted in a total of 16 participants: two participants self-identified as male (12.5%) and 14 as female (87.5%); six participants were students at public high schools (37.5%) and 10 at private high schools (62.5%); a total of 10 students (62.5%) were in Grade 9 and the other six students (37.5%) were in Grade 10 (see Supplementary Table A).

### 2.3. Procedure

All the interviews were held online and *via* Lookback's interview moderation platform^2^ (Lookback Group Inc., Palo Alto, CA). The allocated time for each interview was announced to be between 30 and 50 min, depending on how the discussions would unfold; however, almost all interviews lasted between 30 and 40 min except for those adolescents who answered questions pertaining to both digital and non-digital mental health resources, for whom the interviews lasted close to 50 min.

The interviews were conducted by one principal interviewer and one notetaker. The research team deemed that having a notetaker present during each interview was necessary. Due to ethical considerations and respect for the privacy of the adolescent participants, capturing only the audio of interviews was to be allowed. An important role of the notetaker^3^ was thus to look closely for and to detail contextual and non-verbal cues (such as a change in facial expression, posture, or body language); these cues were very important for uncovering what was not explicitly expressed by participants or remained unknown to them (20). In addition, due to the semi-structured and exploratory aspects of the interviews, and the sensitivity of the subject, the notetaker was instructed to intervene whenever a key probing opportunity might be missed and not used by the principal interviewer, or whenever nonverbal cues could indicate that the existing topic of discussion should be abandoned in favor of another topic.

All the participants were asked to give their consent—a written consent prior to the day of interview and a verbal confirmation of their consent at the beginning of interview—for participating in our study. As planned in our semi-structured interview guides, during each individual interview and from each participant, open-ended questions were asked which followed data gathering techniques that are commonly practiced in user research and Design Thinking methodology (21). As the starting point, the aim of our questions was to understand “when”, “why”, and “how” each student had used either a specific aspect of the chosen digital mental health solution (for the main participant groups) or a digital/non-digital mental health resource (for the supporting groups); then, further elaboration and clarification was requested from each participant regarding what they liked and disliked about their experiences; and lastly, participants were asked what they believed to be the shortcomings of each aspect of their experiences, and what their recommended improvements were for addressing them. Participants who had used non-digital resources were also asked what they perceived or believed digital mental health resources lack compared to non-digital ones.

To better answer our two research questions, for the main participant groups, specific topics and aspects were selected to be the focus of the open-ended questions. These aspects and topics comprised (a) the learning materials and activities within the programs in which the users had participated; (b) the relevance and depth of the offered themed programs and activities; (c) the individual dashboards and overall leaderboards; (d) the announcements (general and program-specific); and (e) the gamified aspects of the web-based platform. Since it was intended that our discussions encompass as many factors as possible pertaining to user experience of this specific solution, discussing all these topics and delving deeper into each one helped us better investigate adolescent students’ perceived experiences, as well as their expectations and desires when using the platform, its programs and features.

### 2.4. Data analysis

As planned, our interview findings from both main and supporting participant groups were to be combined so that more credible evidence and more insights into user engagement factors for DMHI programs could be discovered. For this purpose, a similar procedure was developed for analyzing all the conducted interviews. We adopted the analysis procedure that is commonly employed in user research and in Design Thinking methodology's “Define” phase (21); we closely followed Ulrich & Eppinger's (20) recommended qualitative analysis steps for identifying the users’ needs.

The data needed to be prepared to be suitable for our chosen analysis method. First, the transcript for each interview was developed in the language it was conducted—the participants were given a choice between English or French. Subsequently, the pertinent contextual and non-verbal cues and insights, which were captured by the notetaker were added. The transcripts were then anonymized to protect the privacy of the individuals. On completion, segments of each transcript corresponding to specific participant group (frequent users, infrequent users, digital users, and non-digital users) were assembled into a corresponding word-processing document (i.e., each participant group had a separate document).

Our analysis procedure started after the data had been prepared. We employed an inductive approach in which each sentence within the transcripts (whether it was expressed by the user or added by the notetaker) that represented a user need would be transformed into one or more phrase(s)—as some sentences could represent more than one user need—representing the need(s) from user's point-of-view (i.e., in the format of statements such as “I would like to be able to set clear expectations before using a mental health application.”).

After transforming all the transcripts into phrases that represented user needs, first, redundant phrases expressed by the same participant were omitted. Similar phrases within a participant group were then identified and analyzed as follows: the most representative phrase between similar needs was retained and the count of similar phrases was appended to it; the other similar phrases were then discarded. However, if no single phrase could represent all other similar phrases, this above procedure would not apply. Instead, a new phrase was generated to represent a theme shared among similar needs; its component phrases were then listed *via* bullet points underneath. Using this thematic analysis along with the number of occurrences per each theme helped us answer the first two research questions (RQ1–2), i.e., by pinpointing some of the most important factors related to user experience of digital mental health solutions that could influence the main participants’ paths toward a high or low level of engagement with school-based DMHI programs.

To analyze the data for answering the third research question (RQ3), all of the separately gathered data needed to be combined so they could be analyzed together. To this end, first, phrases from each of the participant groups were copied into an online whiteboard platform, inside separate text boxes. Subsequently, all the text boxes were color-coded to distinguish to which participant group they belonged. Next, all the phrases were combined together as follows: (a) all phrases with no assigned counts or bullet points (which showed they were a repeated or shared need among a specific group) were discarded if they bore no similarity to each other; (b) phrases that shared similarities with each other were categorized together; if one phrase represented all the others within a category, it was retained; however, if no phrase represented all other similar phrases, a more representative one was generated and retained; and (c) in each category, similar phrases were added *via* bullet points underneath the retained phrase, and the count of similar ideas was appended to it (while taking into account the counts that were previously associated with the similar phrases).

In order to properly answer the third research question, a prioritization process was developed. First, phrases were ordered based on the number of occurrences (count of similarities plus the number of bullet points associated with each phrase). Then, the needs with the most urgency were identified; the weight or priority of each urgent need should have been acknowledged either by the adolescent participants themselves (explicitly or implicitly), or by the research team (according to the topic's sensitivity and the gravity of its positive or negative effects). By keeping solely the phrases that had the most occurrences and the most urgency, the user needs that were most likely to influence the users’ experiences and engagement levels with DMHIs were uncovered. Subsequently, whether these user needs could be categorized as a user expectation or an additional desire associated with using a digital mental health solution was investigated. *User experience* is defined by the International Organization for Standardization (ISO) as the “users’ perceptions and responses that result from the use or anticipated use of a system, product or service” (22), hence discovering what the adolescents expect (i.e., perceive as a digital mental health solution's “must have”) or additionally desire (i.e., perceive as a digital mental health solution's “nice-to-have”) to be addressed *via* their experiences with DMHI programs was a key step in our analysis: user expectations and desires can directly influence the perceived user experience of school-based digital mental health solutions.

To better describe and classify the identified users’ expectations and additional desires from digital mental health solutions, two models were used. The first model that was used and adapted to our context was McQuivey's *four fundamental human needs* model, in which the fundamental needs of “comfort”, “connection”, “variety”, and “uniqueness” are claimed to be the factors affecting the individual behaviors of digital users (23, p. 61). This model was chosen as it could help explain what underlying needs motivate adolescent users to expect, or to additionally desire, to have access to certain features or functionalities, to receive certain benefits in using digital mental health solutions. In our adaptation, and based on the links found to our findings from the interviews, we define (a) *comfort* as the user's need to remove complexity, ambiguities and any sources of stress that user experience of digital mental health solutions could bring; many instances similar to this definition were found within our findings, such as our participants’ demands for psychological safety and ease-of-use; (b) *connection* as the user's sense of belonging with their peers, and to feel part of a community; needs similar to these were found to be of great influence in how our participants judged the usefulness of the digital platforms they were using; (c) *variety* as the user's need for anticipating novel experiences, untapped possibilities and diversions from boredom; these underlying needs were found to be felt by our participants when demanding or wishing for more personalization, customization and gamification within digital solutions; and (d) *uniqueness* as the user's need for individuality, self-acceptance, and personal development; needs resembling these were found to be the drivers behind our participants’ expectations and desires for opportunities for improving their self-efficacy, self-reflection, and knowledge (23, p. 61–65).

Lastly, Goodhue & Thompson's *Technology-to-Performance Chain* (TPC) model (24) was used to investigate how each user expectation and desire could be addressed *via* the individual, the technology, or the task characteristics; according to TPC model, technologies could lead to performance impacts at the individual level when utilized by the individual users and fit the user task that they support (i.e., achieving a task-technology fit). Our aim with this last step of analysis was to present how the developers of digital mental health solutions could target users’ expectations and desires through (a) technology characteristics, meaning the aspects or functions related to the systems, hardware, software, training, user support services, etc. (24); (b) task characteristics, i.e., aspects related to the physical and cognitive actions and processes required from individuals who utilize the technology in their environments (25); or (c) adapting or extending their technology and task characteristics to become compatible with individual characteristics of the users, such as their familiarity and experiences with the technology or the tasks it supports, their motivations to utilize the technology or to perform a task, etc. (24).

## 3. Results

### 3.1. Key themes emerged for the main participant groups

In this section, our key findings for the main participant groups, each derived from the themes that emerged after organizing the user needs based on their frequency of repetition and urgency, are presented. Also, the answers to our first two research questions are presented when discussing the key themes found per each user group.

#### 3.1.1. Themes found for frequent users

By performing a thematic analysis of our interviews with the users who had a considerably high engagement level with DMHI programs, their main expectations and additional desires from using universal school-based digital mental health solutions were identified. In other words, these expectations and desires are the main factors driving the users to better engage with DMHI programs (i.e., the answer to RQ1). Below we present these factors (labeled H1–4) together with key quotes from the interviews with this specific user group:

**H1:** Adolescent users demand to be able to adapt the programs and activities to their schedules and realities: **(A)** users expect to be empowered with the ability to scale up, pause or scale down their progress through adjusting the speed, volume, and difficulty level of their activities, where applicable. All the frequent users (4 out of 4) claimed they were willing to perform more activities when they had less homework and were less busy; however, they preferred having no time-consuming activities during the final weeks of the schoolyear, as they would have neither time nor a proper focus for anything other than their final examinations. By demanding flexibility, participant P3 (female, aged 15–16) expressed: *“For me, one activity per week is the right number when I am busy with exams, for example, [if only] it can be carried over to the next week!”*; and **(B)** users expect to be presented with learning and activities that would have real-life application for them. Half of the users (2 out of 4) expressed they required realistic applications for their learning activities, with recommended steps for applying them in practice. About the instructions on how to complete certain activities, participant P3 insisted: *“I try to follow what they say, but sometimes, some challenges are hard to achieve. They are things you don't normally do!”*.

**H2:** Adolescent users demand receiving incentives for participation in DMHI programs: **(A)** users expect to feel connected with others and encouraged through social connections within the digital environment. A majority of the users (3 out of 4) strongly felt that having a platform to discuss with peers or their friends about their shared activities and readings could be very motivational. In particular, participant P2 (female, aged 15–16) emphasized the importance of having a mental health discussion forum with her peers: *“There are no Quebec websites, to my knowledge, where you can talk with teenagers that lived through same things [problems], understand how they deal with it … preferably anonymously, even moderated, in case someone was sharing dangerous or suicidal thoughts!”*; **(B)** users desire to receive timely and clear announcements about upcoming programs and activities. Regarding announcements of the upcoming learning topics and activities, while one user welcomed the surprise effect of not knowing about them in advance, half of the users liked to be informed beforehand so they could anticipate them or get excited about them. As participant P4 (female, aged 14–15) pointed out: *“It would be nice to have a schedule to know when and what activity is coming out instead of just waiting to see [new announcements] in an email”* [translation from French]; and **(C)** users desire to receive motivational newsletters and emails. Half of the users felt that the platform's newsletters and emails should be accompanied by a form of uplifting or motivational messages since having announcements alone was often not enticing enough for participating in the current or upcoming weekly- or monthly-themed activities. Although a frequent user, participant P4 explained why she was not at first eager to follow the DMHI programs: *“The email invitations did not seem appealing [to me]… they were vague and felt impersonal!”* [translation from French].

**H3:** Adolescent users demand having access to comprehensive information within the digital environment: **(A)** users expect to be provided with access to professional help. Most users (3 out of 4) felt they should learn how to receive help and have instant access or link to professional support, should the need arise. Through asking a rhetorical question, participant P1 (female, aged 14–15) drew attention to the importance of this matter: *“Seeking help and seeing a psychologist is still a taboo, but having problems is not?”* [translation from French]; **(B)** users desire to have access to in-depth learning opportunities surrounding mental health topics or to know how they can learn more about them. Half of the users wished to have more comprehensive information about the mental health-related topics presented within the programs, and if not possible, learn how to acquire the knowledge on their own. Although participant P4 expressed that she did *“enjoy learning about topics that can affect us as an individual, like self-confidence or relationships”*, but as she often wanted to obtain more in-depth information about the subjects, she resorted to external online research: *“I will check on the internet, then type in the topic and continue to learn about the subjects that interest me … but often they [sources] are not accessible or simple [to use]”* [translation from French]. A similar concern was also voiced by participant P2 as she desired to *“have information on different cultures, ethnic groups […] and more playful subjects, like how young people listen to music, [enjoy] popular activities and books that help them”*, but she finds herself *“searching the internet sometimes but don't know where to look!”*; and **(C)** users desire to learn more about the sensitive topics that are relevant to them (such as addiction, abortion, etc.). The offered DMHI programs were believed to only scratch the surface of sensitive and taboo subjects rather than providing a thorough and detailed examination of them which half of the users would have preferred. As participant P1 clarified: *“The programs helped me in a way because they taught me about addiction, love, etc., but I don't know if they really **helped** [emphasis added] me”* [translation from French].

**H4:** Adolescent users want to practice self-reflection and introspection through different topics and activities: **(A)** users desire to have access to a variety of activities and learning materials that encourage self-reflection. Half of the users acknowledged that learning about different topics, and having access to various contents, was nurturing as they allowed them to examine themselves through different lenses and ponder on certain subjects and questions for the very first time. As participant P2 acknowledged about having articles to read, followed by introspective activities (such as quizzes): *“They are very useful. I like to read them to have a better general knowledge, [also to] send the articles to others around me, [to] answer the questions! I’d love that if there were more [questions]!”*; and **(B)** users desire to have access to self-assessment and self-reflective questionnaires and tests, as half of the users claimed they helped them reflect and understand more about themselves. Particularly, participant P4 acknowledged why and how the questionnaires are helpful: *“I like questionnaires. They allow us to be more involved. It's a kind of feedback about ourselves: how we feel, how we act in certain circumstances … these [types of] questions are deeper compared to what we, by ourselves, would think about”* [translation from French].

#### 3.1.2. Themes found for infrequent users

Through thematically analyzing our interviews with the users who had a considerably low level of engagement with DMHI programs, their main expectations and additional desires from using universal school-based digital mental health solutions were identified. In other words, these expectations and desires are the main factors whose absence have led and could lead the users to disengage from DMHI programs (i.e., the answer to RQ2). In the following summary, we present these factors (labeled L1–4) together with key quotes from the interviews with this specific user group:

**L1:** Adolescent users want to have psychological safety when using a digital mental health solution: **(A)** users expect to feel free to use the digital solution and follow its programs. The solution's features and facets (including those of its themed DMHI programs) were deemed not inviting and personal enough according to half of the infrequent users (2 out of 4) as they saw the programs and activities more like their homework rather than exercises to help them. As participant P6 (female, aged 14–15) explained why at some point she decided not to use the mental health solution any longer: *“I stopped using it since it was not targeted at me and I had to do things without understanding! I don't like doing something without knowing why! It was just like an assignment [to me]!”* [translation from French]. Participant P8 (female, aged 15–16) was the other participant who was vocal in her similar criticism as well: *“I have enough homework already, [and] don't want to have another with an app on top of it!”*; and **(B)** users expect to feel at ease when they cannot follow the DMHI programs. Half of the users demanded having the choice to not get included within comparison and competitive features of the platform such as leaderboards; they felt an immense pressure after discovering they were being compared with others, as they believed their varying availabilities, challenges and pressures in daily life did not let them pursue the programs regularly or with the consistency that they wished. After admitting to such conflicts, participant P6 suggested: “*Instead of the scoreboard, which is useless and stressful, there should be a section on meditation, etc. There could be sections for relaxing!”* [translation from French]. Although not opposed to the idea of having performance statistics within a digital mental health platform, participant P5 (female, aged 14–15) requested a greater peace of mind by having the option *“to show the leaderboards only between friends”* [translation from French], instead of a global one that would include every user from all the participating schools.

**L2:** Adolescent users want access to personalized contents and activities: **(A)** users expect to know if the program would be able to help them, and how. A majority of the infrequent users (3 out of 4) felt the programs’ activities were not necessarily targeted at them and they had to do them without understanding what their benefits were or how they could receive them. As participant P6 ascertained: *“If these [kinds of] programs can explain how it can help young people in the first place, surely young people will relate to it more”* [translation from French]. In addition, participant P7 (female, aged 14–15) further elaborated—sarcastically—on why she felt that something was lacking: *“I remember a text [titled] ‘what is love?’, with examples of couples … I don't learn anything [from it]; I don't feel challenged! I didn't think: Oh, I learned about love, I should now see what it means in my life!”*; and **(B)** users expect to have the ability to choose from a range of topics and activities and to select the ones that would be (more) appropriate or applicable to them. Half of the users required, but were not given, the ability to choose the activities they wanted and the ones they did not want to participate in for the duration of themed programs. As participant P6 articulated how she wanted to use the solution: *“I would have liked it to be separated into sections, so I could pick only the content that is relevant to me only! [I would suggest] keeping things simple, with keywords!”* [translation from French].

**L3:** Adolescent users want to have incentives to participate: **(A)** users expect to have access to a platform where they could discuss with other peers. All the infrequent users (4 out of 4) required the ability to communicate with other young people through the digital platform, especially with other students or friends who experienced the same things. Participant P5, in particular, was adamant about this request: *“Talking with other people of the same age who have been through the same things as you, that [emphasis added] is better than talking with adults”* [translation from French]; and **(B)** users desire to receive follow-up on past activities and learning they had completed in order to feel or realize how much they have grown since the last time, and to continue their progress. Half of the users were discouraged in continuing to use the programs because they felt their prior efforts were not accounted for. Not only did participant P6 express that: *“There was no follow-up. There should be workshops in class or discussions to follow up [on activities], but not too often!”* [translation from French], but also participant P7 voiced her concern about the possibility that even the same-week activities could be overlooked by students with busy schedules: *“If the emails are opened at a moment that I’m at school or somewhere that I can't read it properly, I might totally forget to go back to that week's activity later!”*.

### 3.2. Emergent themes from combining data from all participant groups

**Figure 1:**
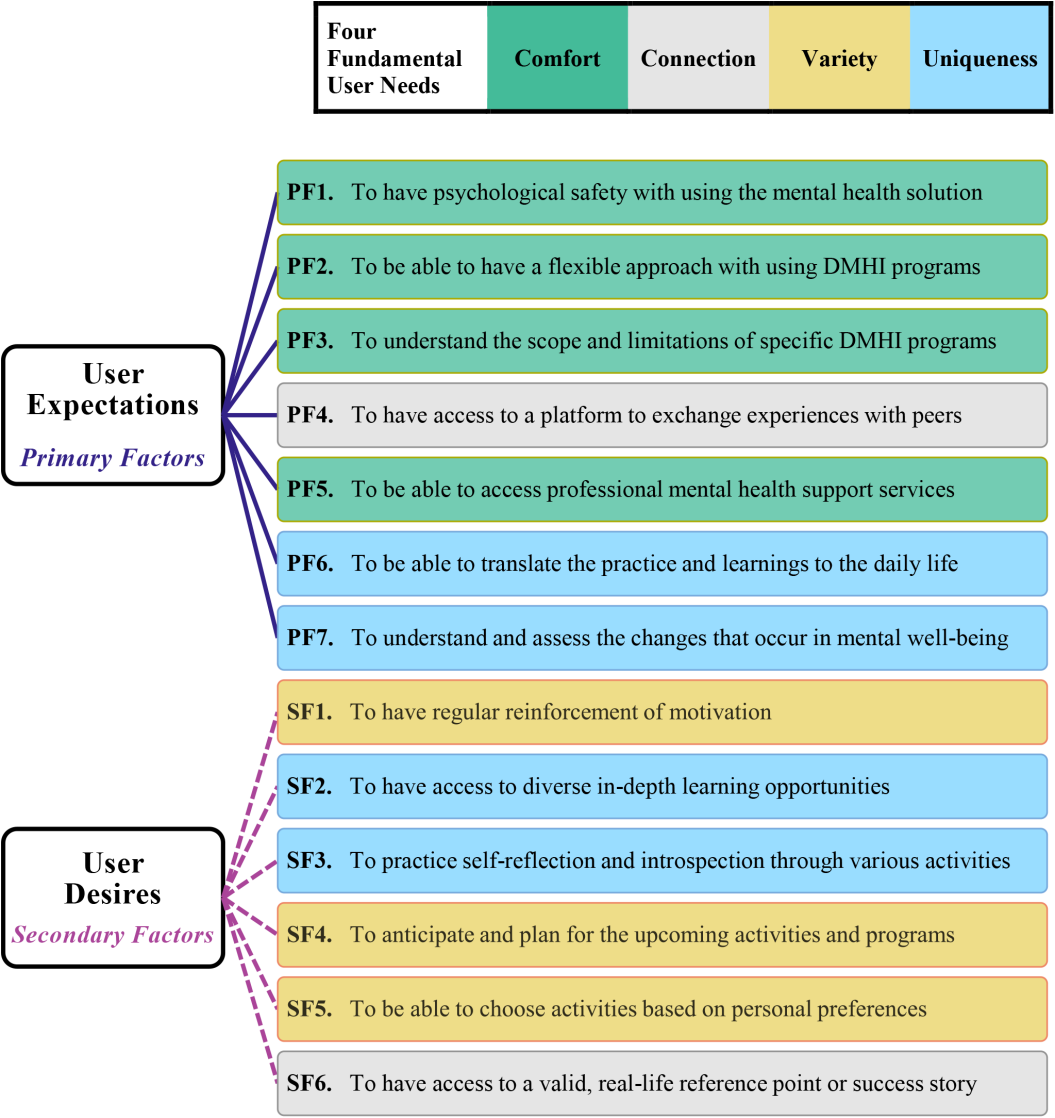
Primary and secondary factors facilitating user engagement with DMHIs. *Note*. The color scheme of each primary factor (PF#) and each secondary factor (SF#) corresponds to the color scheme representing the four fundamental user needs.

After analyzing the combined data from all 16 participants interviewed in our four participant groups, the most important user expectations, and the most prominent additional desires of users associated with digital mental health solutions were identified. The identified user expectations, which we recognize as the *primary factors* related to user experience of DMHIs that facilitate engagement (labeled as PF1–7), and additional user desires, which act as *secondary engagement factors* with DMHIs (labeled as SF1–6) were then linked to our adapted concepts from the four fundamental human needs model. The result, in other words the answer to the central research question of our study (i.e., RQ3) is presented visually *via* Figure 1; the same color scheme is used for our findings and the model for the purpose of clarity.

As apparent from Figure 1, all the user needs concerned with the users’ pursuit of *comfort* fall into primary factors of engagement, since they were ranked among the most important and urgent needs of the users in using school-based DMHI programs: having psychological safety (PF1) and a flexible approach (PF2) in following the DMHI programs and when needing professional support (PF5), also understanding what they can learn from and can accomplish by following a specific program (PF3). Furthermore, user needs concerned with their search for *variety* fall into secondary engagement factors since their fulfillment was considered to be very desirable and motivating, but not an absolute necessity: having regular reinforcement of intrinsic and extrinsic motivation (SF1), building anticipation of possibilities in the future (SF4), and being able to progress in the programs while having the freedom to choose from activities based on personal preferences (SF5).

As can also be observed from Figure 1, striving for connections was not only the underlying reason behind one of the most repeated requests among the main participant groups (i.e., the identified factors H2A and L3A), but it also falls into both primary and secondary user engagement factors with DMHIs: the users’ need for *connection* not only accounts for their expectation of having access to a peer discussion platform (PF4), but it also represents their desire to access or follow real-life examples as their reference points (SF6), in pursuing the betterment of their mental health. Moreover, the user needs that were concerned with appreciating the individuality and *uniqueness* of oneself were (a) responsible for the last two primary engagement factors: being able to adapt the mental health-related activities to their daily life (PF6), being able to understand the changes that can occur in their mental well-being, and to learn how to assess these changes (PF7); and (b) among the most important secondary factors: the need for having a variety of opportunities for self-improvement (SF2), as well as for self-reflection and introspection (SF3).

**Figure 2:**
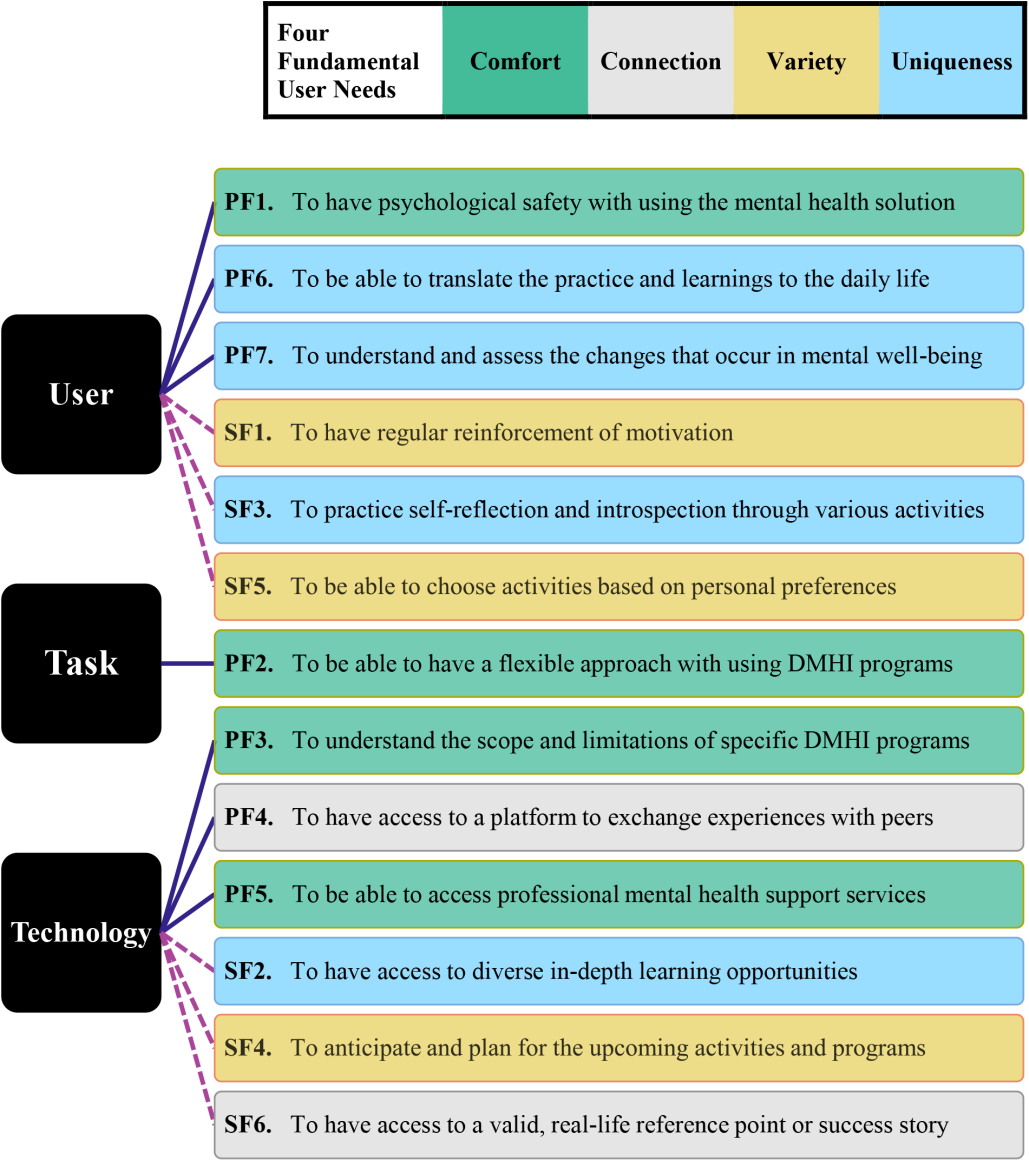
Integrative diagram to inform the user experience of universal school-based DMHIs. *Note*. The color scheme of each primary factor (PF#) and each secondary factor (SF#) corresponds to the color scheme representing the four fundamental user needs.

To inform the user experience of school-based DMHI programs, Goodhue & Thompson's TPC model (24) was integrated to our findings so it could be determined which dimension between technology, task, and individual user characteristics should be targeted for addressing each of the identified engagement factors. In Figure 2, the result of this integration is represented after reorganizing the engagement factors based on their order of importance according to the following logic: (a) the expectations (i.e., primary engagement factors) precede the additional desires (i.e., secondary engagement factors), as they were the least of the users’ demands that should be met; and (b) a lower number (starting from 1) represents greater importance in each user expectation and additional desire, as they were labeled in descending order of occurrence and urgency. The breakdown of our findings and practical implications for each of the primary and secondary engagement factors are detailed in Tables 1, 2, along with our recommended approach for addressing each factor.

**Table 1:** Implications of the identified primary engagement factors and our recommendations.

Factor	Underlying user need	TPC dimension	Implication of our findings and our recommended approach
**PF1**	Comfort	Individual user	This expectation should be addressed by adapting and extending the programs and activities to become compatible with the users’ motivations to use the digital solution, while respecting the following demands: (a) users expect to feel comfortable using the solution by doing so without feeling the pressure of commitment or obligation and thus feel invited and welcome to continue their progress whenever they would log back in; (b) users expect to be able to view their participation in the programs as a normal activity intended for everyone (as opposed to feel as if the programs are targeted for people who are not normal); and (c) users expect to have the choice to give permission for using their data for comparison purposes.
**PF2**	Comfort	Task	This expectation should be addressed *via* aspects related to physical and cognitive actions and processes that are required from the users when using the solution, while respecting the following demands: (a) users want to feel comfortable using the digital solution at their chosen times, environments and conditions (i.e., not being bound to follow the programs in specific time or place, such as a classroom), independently and by themselves without the presence of an instructor or a guide; and (b) users want to be able to adapt the programs and activities to their schedule (especially considering the busy examination period) and their reality (*via* recommendations that could be applied to real life).
**PF3**	Comfort	Technology	This expectation should be addressed through aspects related to the solution's characteristics by removing ambiguities, as much as possible, surrounding what users can expect and what they cannot expect from using the solution, its programs, and resources. The ambiguities would be minimized if the users are provided with (a) a guidance and support services (e.g., help and documentation) to get a clear understanding of how to use the solution, programs and resources while dispelling incorrect preconceived notions about them; and (b) to understand what benefits they can expect from the programs and when can they expect to see them after following the recommended steps for their completion.
**PF4**	Connection	Technology	This expectation should be addressed *via* aspects related to the technology characteristics by providing a discussion platform where the users: (a) feel safe and supported to connect with their peers and follow their discussions; and (b) feel empowered and encouraged to share their personal experiences and difficulties with others.
**PF5**	Comfort	Technology	This expectation should be addressed *via* aspects related to the technology characteristics, by providing easily accessible mental health support services to the users. The users should be provided with a simple, hassle-free way to contact an external support team or professional if personal or program-specific problems arise during or outside the time and place where they would use the solution; therefore, the support personnel should also be able to answer questions about the program(s) and activities and to guide the students in completing them.
**PF6**	Uniqueness	Individual user	This expectation should be addressed through adapting and extending the programs and activities to become compatible with the users’ motivations to perform mental health-related tasks, by respecting the following demands: (a) users expect to be able to integrate mental health-related activities and techniques into their daily routines (i.e., perform activities in a place they already go, during the availabilities and with the accesses they already have); and (b) users expect their learnings (concepts, techniques and methods, etc.) can be implemented and put in practice in their day to day lives.
**PF7**	Uniqueness	Individual user	This expectation should be addressed through adapting and extending the programs and activities to become compatible with the users’ motivations to use the solution, by empowering the users to: (a) understand the changes that can occur in their mental and physical health (due to stress, anxiety, depression, etc.), their causes, and how to alter or prevent them; (b) understand what they can change (either by themselves or receiving professional help) and what cannot be controlled; and (c) track progress and changes that have occurred in their mental health since the start of their participation in the program.

Each factor (PF#) named below represents a primary engagement factor as described in Figures 1, 2.

**Table 2:** Implications of the identified secondary engagement factors and our recommendations.

Factor	Underlying user need	TPC dimension	Implication of our findings and our recommended approach
**SF1**	Variety	Individual user	This additional desire could be addressed through adapting and extending the programs, activities, learning and supplementary materials to become compatible with the users’ motivations for performing mental health-related tasks, by empowering the users to: (a) cultivate internal sources of motivation and avoid external motivations focused on competition; (b) regularly benefit from the supporting learning activities and practices; (c) understand how the intangible benefits of partaking in the program's activities can be felt; and (d) feel their past efforts are accounted for and would be pursued further.
**SF2**	Uniqueness	Technology	This additional desire could be addressed *via* aspects related to the technology characteristics, by providing learning opportunities that: (a) are presented and repeated through different methods (integrated within videos, readings, etc.); (b) deliver or instruct on how to acquire in-depth education on mental health topics; (c) can evolve and advance over time (with the user's or the semester's progress); and (d) offer easy-to-understand lessons and readings on relevant sensitive subjects (such as bullying, addiction, or racism).
**SF3**	Uniqueness	Individual user	This additional desire could be addressed through adapting and extending the programs’ materials to become compatible with the users’ motivation for learning about themselves and the aspects unique to themselves *via* questionnaires and quizzes, learning activities and practices that encourage self-reflection and introspection.
**SF4**	Uniqueness	Technology	This additional desire could be addressed *via* aspects related to the technology characteristics, by providing timely and clear communications surrounding the upcoming activities and programs, also the required effort to achieve the desired outcome from the learnings and practices so that the interested users can accommodate their plans for them.
**SF5**	Variety	Individual user	This additional desire could be addressed through adapting and extending the programs, activities, learning and supplementary materials to become compatible with the users’ personal characteristics and preferences such as through providing adjustable settings for difficulty, usage frequency, and concentration levels required for completing any program.
**SF6**	Connection	Technology	This additional desire could be addressed through technology characteristics, by providing: (a) a dedicated forum where the users can understand how other peers are experiencing the same program; (b) inspiring success stories and anecdotes with which users can learn about experiences of others who overcame the difficulties that were similar to themselves; and (c) access to qualified peers who could act as users’ mentor or guide for the program.

Each factor (SF#) named below represents a secondary engagement factor as described in Figures 1, 2.

## 4. Discussion

### 4.1. Overview of key findings

By conducting exploratory user research, comprising qualitative interviews with different groups of adolescents who were either actual users or potential users of DMHI programs, the most important (or primary) and the additionally desirable (or secondary) factors that could lead the adolescents to better engage with school-based DMHI programs were identified.

The primary factors (labeled **PF1–7**), those related to the user experience of digital solutions that directly influence users’ engagement with school-based DMHIs, were identified, in order of their importance and priority, as follows: (a) having psychological safety with using the digital mental health solution (**PF1**); (b) being able to use DMHI programs with a flexible approach (**PF2**); (c) understanding the scope and limitation of DMHI programs (**PF3**); (d) having access to a platform to exchange experiences with peers (**PF4**); (e) being able to access professional mental health support services, when the needed arises (**PF5**); (f) being able to translate and anchor practices and learning activities into daily life (**PF6**); and (g) being able to understand and assess the changes that could occur in one’s mental well-being (**PF7**).

Moreover, the secondary factors (labeled **SF1–6**) related to the user experience of digital solutions that could facilitate a higher level of user engagement with school-based DMHIs were identified, in order of importance and priority, as follows: (a) having regular reinforcement of motivation (**SF1**); (b) having access to diverse in-depth learning opportunities (**SF2**); (c) practicing self-reflection and introspection through diverse reading, learning, and other activities such as questionnaires (**SF3**); (d) anticipating and planning for upcoming programs and activities (**SF4**); (e) being able to choose activities based on one’s personal preferences (**SF5**); and (f) having access to valid, real-life reference points and success stories (**SF6**).

### 4.2. Theoretical contributions

This study contributes to digital mental health, mental health intervention, and user experience literature by (a) exploring the factors that help explain why adolescent users become more engaged with certain universal school-based DMHI programs than with others; and (b) identifying how the experience of using a specific universal school-based DMHI program could become more engaging for adolescents. To our knowledge, no recent qualitative exploratory research had been conducted exclusively with adolescents of high school age while seeking to identify factors that influence their engagement with school-based DMHIs in non-treatment-focused settings (i.e., DMHIs not primarily used as a means for mental health treatment, counselling, or therapy for at-risk individuals or groups).

Recent and concurrent research efforts have been made that either (a) investigate acceptability of treatment-focused school-based DMHIs among high school students, such as O’Dea et al.'s study (26); (b) explore the adolescents’ use and preferences in treatment-focused digital mental health, like Aschbrenner et al.'s study (27); (c) explore the perspectives and preferences of young people including—but not focused on—adolescents regarding school-based digital mental health services through conducting a pilot study, for example, Garrido et al.'s study (28); (d) explore the experiences of young adults above high school age with non-school-based digital mental health applications, such as Borghouts et al.'s study (29); (e) explore the barriers and facilitators of engagement with non-school-based DMHIs for adults above high school age, like studies conducted by Borghouts et al. (30) and Auster-Gussman et al. (31); or (f) discuss the potential benefits and risks of using digital mental health for adolescents, such as works by Cefai et al. (32), Yilmaz et al. (33), and Wies et al. (34). The mentioned studies have been very important and encouraging due to their focus in such sensitive topics and their goals of advancing the body of knowledge pertaining to DMHIs or digital mental health resources in general. None, however, had particularly concentrated on non-treatment-focused digital interventions in high schools while exploring the perspective of their actual end-users or investigating user engagement factors with DMHIs.

Nevertheless, it is important that we acknowledge and compare our approach and results with a few studies that were more relevant since they shared approaches or exploratory goals similar to ours, namely the works by Torous et al. (35), Babbage et al. (36), and Szinay et al. (37).

Earlier, in 2018, Torous et al. (35) had theorized that poor usability, lack of user-centric design, privacy concerns, lack of trust as source mental health information, and lack of emergency measures were the five factors most responsible for low engagement with mental health apps. This theory came from a selective narrative review with the goal of finding themes among the reasons for low engagement with mental health smartphone apps; the study was primarily focused on the use of apps for personal or clinical treatment purposes and did not include studies about school-based interventions. On the contrary, our study had a much more specific focus: it was focused solely on adolescents of high school age and on non-treatment-focused DMHIs; also, it was specifically concerned with school-based DMHIs as its main target. Considering that DMHIs in high schools introduce additional variables and concerns due to their specific restrictions, pre-defined target users and objectives, it is not surprising that none of Torous et al.'s five responsible factors were among those identified to be responsible for the lower-than-average engagement level of our participants; instead, these factors were related to users’ psychological safety, and the applicability and usefulness of the digital solution for the user, all being concerns particular to the age group and the context of school-based DMHIs.

Also published in 2018, Babbage et al.'s qualitative study (36), which aimed to explore adolescents’ desires in using nonclinical digital tools for well-being self-management was similar in its data collection and analysis methods to ours (i.e., semi-structured interviews with adolescents of high-school age), but with one major difference: the participants had not used any digital solutions to manage their well-being prior to the interviews. Our study, on the other hand, was designed and envisioned to cover more ground by including users with extensive experiences (frequent users); moderate experiences (users of non-school-based digital resources); and limited experiences (infrequent users) with nonclinical digital mental health tools; also, potential users who, unlike Babbage et al.'s nonuser participants, had experiences with non-digital mental health resources with purposes similar to well-being self-management. Despite encompassing participants with different degrees of experience with digital mental health resources, some of our study's findings from the main participant groups (i.e., users with extensive or limited experiences) were found to have close similarities with Babbage et al.'s findings from their nonuser participants. These specific similarities are: the expectation of adolescents to receive professional help in case of mental health emergencies, where Babbage et al.'s *Theme 3.2* from *facet-based* themes (i.e., “providing information and direction for further support”) matched our *Theme H3A*; and Babbage et al.'s *feature-based* themes, *Theme 5* (“flexibility in choice and resources”) and *Theme 6* (“enabling engagement with others”) closely resembled our respective findings: *Theme L1*, and both *Themes H2A* and *L3A*. These similarities between findings from our main participant groups and Babbage et al.'s findings from their nonuser participants—which could also be found when comparing their results with our findings after combining both the main and supporting groups—suggest that our decision to include the supporting groups of participants was indeed helpful; this not only provided us with more evidence and insights into adolescents’ experiences with mental health resources, but also increased the credibility of the user engagement factors that we identified to be facilitators of adolescents’ engagement with DMHIs.

More recently, findings from Szinay et al.'s (37) systematic review of digital behavior change intervention studies, which was focused on the uptake and engagement surrounding health and well-being smartphone apps, had some similarities with our qualitative study. Although Szinay et al.'s review was concentrated on the adult population (aged 18 and above), and only included studies with adolescents aged 16 and above if at least 70% of their participants were adults, a number of factors influencing engagement were shared between our studies. The identified *knowledge* themes of “User guidance” and “Health information” from Szinay et al.'s study closely resembled factors discovered for our *Theme H3*; their *Environmental context and resources* theme of “Personalization to needs” resembled our *Theme L2*; and lastly, their *Social influences* themes of “Health practitioner support” and “Community networking” respectively matched *Theme H3A*, and both *Themes H2A* and *L3A*. Nevertheless, the “Social competition” theme found from Szinay et al.'s study directly contradicts our *Theme L1B*; unsolicited comparisons and competitions were recognized by our adolescent participants as sources of immense psychological pressure. This contrast was however to be expected since the focus of our study was on the use of DMHIs in school settings and by adolescent users who have life pressures, challenges, and mental barriers that, arguably, are vastly different from older age groups. In our study, to have psychological safety—which contradicts the social competition theme identified in Szinay et al.'s study—was recognized as the users’ main priority and the most important factor driving the adolescents’ engagement with DMHIs (*PF1*).

### 4.3. Implications for practice

To help inform developers and designers of school-based DMHIs in designing the user experience of digital mental health solutions, theoretical approaches (i.e., the TPC, and four fundamental human needs frameworks) were used that assisted us in investigating which capabilities, features and functionalities within digital mental health solutions should be focused to address the identified engagement factors. Since our recommendations for addressing the engagement factors are derived directly from analyzing our interview data, they can help increase the likelihood of the user experience of digital mental health solutions meeting the expectations and desires of adolescent students, and in doing so, facilitate users’ optimal level of engagement with universal school-based DMHI programs. The implications of each engagement factor and our recommended approach to address them are detailed in Tables 1, 2.

### 4.4. Limitations and research avenues

The results of our study must be seen in light of the limitations that it was subjected to, especially due to the sensitive nature of studies on the topic of mental health, particularly with adolescents. In the following paragraphs, we present the major limitations of this study and the avenues we believe should be considered in related future research.

Since our recruitment and data collection processes started during the busiest period of the schoolyear (i.e., a few weeks before the final examination period), students were primarily concerned with upcoming examinations and less likely to check their emails attentively. Hence, the research team was unable to recruit more participants for each group and to have more representative groups of participants. Specifically, for the main participant groups, out of a total of 33 potential participants identified as *frequent users*, only five (15.1%) were male students; in addition, there were also another 33 potential participants for the *infrequent users* group, of which only four responded to the invitations (and after two rounds of sending the invitations), and none were male students, even though they represented 27.3% (nine out of 33) of the sample. Qualitative research opportunities should be pursued in the future with similar focus, goals, and exploratory approach centered on identifying the factors that could influence user engagement with school-based DMHIs. However, the sampling limitations of this research must be addressed by capturing a larger sample of more representative individuals (especially by recruiting a selection of individuals with more variance in their gender identities and their age-groups). In addition, the period in the calendar and schoolyear needs to be mindful of the adolescents’ schedules (i.e., not close to holidays or examination periods, both of which could introduce unforeseeable complications in participants’ schedules and willingness to participate).

Since our potential participants were mostly contacted through their schools’ email address or schools’ newsletters, there were concerns that they—especially infrequent users—might believe they were being questioned for their low activity within the programs and might thus decide against participating in our study or inquiring further about it. The research team aimed to proactively address any such concerns by promoting this study as being completely independent from both the schools and the mental health resources to be discussed. Our findings from the interviews strongly indicated this approach was successful; our participants held no reservations in discussing their honest opinions, preferences, and felt experiences with the digital mental health resources in question. It cannot be ascertained whether having a certain level of trust between the participants and their high schools or the professionals responsible for digital mental health resources had initially influenced the students’ participation, nor can it be objectively investigated or measured whether the level of this trust might have affected users’ engagement with digital mental health resources. Therefore, an exciting and important research opportunity that we propose is to focus on how the level of trust between the students and their high schools or their relevant staff affects students’ engagement levels with universal school-based DMHIs.

Last but not least, many parallels were found between the results of our research and the findings from a systematic review that aggregated the data from studies with similarities to ours (37). However, since a purposive sampling method was used in this study to limit major contextual differences in perceptions, opinions, and experiences of the adolescent participants, which could introduce uncontrollable variables in this type of qualitative research, it cannot be established that replicating our methodology in other contextual settings would result in finding the same engagement factors identified in our study. Therefore, promising avenues for future research reside in constructing a series of qualitative research efforts with the goal of identifying user engagement factors with school-based DMHIs through replicating or encompassing the groups that were targeted in this study. Future studies should capture users with different degrees of experience with non-treatment-focused digital mental health), and each should be set in a different social, cultural, and physical environment, as these factors could shape differences in adolescents’ perceptions, opinions, and experiences (15).

## 5. Conclusion

Through exploratory research on a topic that required urgent attention, this study sought to extend the body of knowledge pertaining to user engagement through uncovering the needs of adolescents—either with or without prior experiences with digital mental health resources—that are targeted for nonclinical school-based DMHIs. Moreover, this study aimed to contribute to practice by helping inform the user experience of digital interventions through ensuring that the expectations and desires of their targeted users could be met. To this end, specific recommendations are put forward to address each identified engagement factor by depending solely on findings from the interviews that were conducted with actual and potential end-users of school-based DMHIs. It is hoped that our findings and recommendations can help increase the likelihood of effectiveness of universal school-based DMHI programs for high school students and can be used by designers and developers in the user experience and human-computer interaction fields in addressing their users’ needs and creating more engaging programs for them. Furthermore, it is hoped that this effort raises more attention towards the topic, in both academia and practice, and also inspire academic researchers to continue to extend this study's findings.

## Data Availability

The datasets presented in this article are not readily available because there are legal reasons involved due to the collaboration with a third-party organization in partnership with our institution, HEC Montréal. Requests to access the datasets should be directed to SS via ss@hec.ca.

## Footnotes

^1^This measure was put in place to comply with Section 21 of the Civil Code of Québec (chapter CCQ, c. 17, s. 2), in which it is stated that only minors aged 14 and above could give consent alone with the condition that a competent research ethics committee would judge the circumstances to justify involving them.

^2^ https://www.lookback.com/

^3^Four volunteer students from our institution who were authorized by REB to participate in our research helped in both notetaking roles and also conducting the interviews.
