# Supplementary material for: Facilitating engagement of universal school-based digital mental health solutions through user experience: A qualitative exploration
Source: Front Digit Health. 2023 Mar 22;5:1040739. doi: 10.3389/fdgth.2023.1040739 (PMC10075357; doi:10.3389/fdgth.2023.1040739)
Supplement: Supplementary file 1 [file Table1.docx]

Supplementary Material

# Supplementary Tables

**Supplementary Table A.** Characteristics of the interview participants.

| **Participant** | **Gender** | **Participant Group** | **Grade Level** | **Age Group** | **School Type** |
| --- | --- | --- | --- | --- | --- |
| **P1** | F | Frequent User | Grade 10 | 15–16 | Public |
| **P2** | F | Frequent User | Grade 10 | 15–16 | Public |
| **P3** | F | Frequent User | Grade 10 | 15–16 | Private |
| **P4** | F | Frequent User | Grade 9 | 14–15 | Private |
| **P5** | F | Infrequent User | Grade 9 | 14–15 | Private |
| **P6** | F | Infrequent User | Grade 9 | 14–15 | Private |
| **P7** | F | Infrequent User | Grade 9 | 14–15 | Private |
| **P8** | F | Infrequent User | Grade 10 | 15–16 | Private |
| **P9** | F | Non-Digital | Grade 9 | 14–15 | Private |
| **P10** | F | Digital | Grade 9 | 14–15 | Private |
| **P11** | M | Digital | Grade 10 | 15–16 | Public |
| **P12** | M | Both Non-Digital and Digital | Grade 10 | 15–16 | Public |
| **P13** | F | Both Non-Digital and Digital | Grade 9 | 14–15 | Private |
| **P14** | F | Both Non-Digital and Digital | Grade 9 | 14–15 | Private |
| **P15** | F | Both Non-Digital and Digital | Grade 9 | 14–15 | Public |
| **P16** | F | Non-Digital | Grade 9 | 14–15 | Public |
